# Giant cells: multiple cells unite to survive

**DOI:** 10.3389/fcimb.2023.1220589

**Published:** 2023-09-05

**Authors:** Shreyasee Hazra, Suman Kalyan Dinda, Naba Kumar Mondal, Sk Rajjack Hossain, Pratyay Datta, Afsana Yasmin Mondal, Pushkar Malakar, Dipak Manna

**Affiliations:** ^1^ Department of Biomedical Science and Technology, School of Biological Sciences, Ramakrishna Mission Vivekananda Educational and Research Institute (RKMVERI), Kolkata, India; ^2^ Institute of Health Sciences, Presidency University, Kolkata, West Bengal, India

**Keywords:** *Entamoeba*, multinucleated giant cells (MGCs), encystation, heat stress, cell fusion

## Abstract

Multinucleated Giant Cells (MGCs) are specialized cells that develop from the fusion of multiple cells, and their presence is commonly observed in human cells during various infections. However, MGC formation is not restricted to infections alone but can also occur through different mechanisms, such as endoreplication and abortive cell cycle. These processes lead to the formation of polyploid cells, eventually resulting in the formation of MGCs. In *Entamoeba*, a protozoan parasite that causes amoebic dysentery and liver abscesses in humans, the formation of MGCs is a unique phenomenon and not been reported in any other protozoa. This organism is exposed to various hostile environmental conditions, including changes in temperature, pH, and nutrient availability, which can lead to stress and damage to its cells. The formation of MGCs in *Entamoeba* is thought to be a survival strategy to cope with these adverse conditions. This organism forms MGCs through cell aggregation and fusion in response to osmotic and heat stress. The MGCs in *Entamoeba* are thought to have increased resistance to various stresses and can survive longer than normal cells under adverse conditions. This increased survival could be due to the presence of multiple nuclei, which could provide redundancy in case of DNA damage or mutations. Additionally, MGCs may play a role in the virulence of *Entamoeba* as they are found in the inflammatory foci of amoebic liver abscesses and other infections caused by *Entamoeba*. The presence of MGCs in these infections suggests that they may contribute to the pathogenesis of the disease. Overall, this article offers valuable insights into the intriguing phenomenon of MGC formation in *Entamoeba*. By unraveling the mechanisms behind this process and examining its implications, researchers can gain a deeper understanding of the complex biology of *Entamoeba* and potentially identify new targets for therapeutic interventions. The study of MGCs in *Entamoeba* serves as a gateway to exploring the broader field of cell fusion in various organisms, providing a foundation for future investigations into related cellular processes and their significance in health and disease.

## Introduction

**Figure 1:**
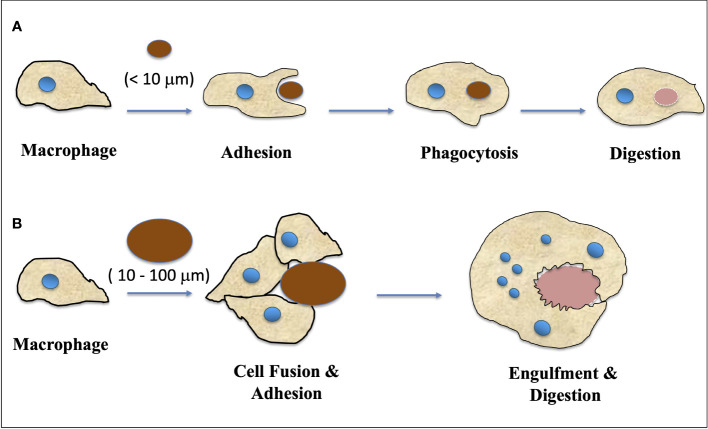
A schematic representation of how macrophage transformed into MGC (Multinucleated Giant Cells). **(A)** Macrophages respond to small fragments and particles (<10 μm in diameter) by internalization via phagocytosis and intracellular digestion. **(B)** When the particle size is larger than 10 μm and smaller than 100 μm, the macrophages fuse together, forming giant cells with multiple nuclei which in turn engulf the particles and digest them. (Adapted from Sheikh et al., 2015).

Fusion of cells and the subsequent creation of multinucleated giant cells (MGC) is a common occurrence in animals, particularly in response to inflammatory reactions such as infections with tuberculosis, HIV, herpes, or foreign bodies (Peterson et al., 1996; Lay et al., 2007; Vérollet et al., 2010; Yamamoto et al., 2019). There are various lineages of cells in the human body, including monocytes and macrophages, that are capable of forming multinucleated giant cells (MGCs) (Orentas et al., 1992; Brooks et al., 2019). There are several types of giant cells that have been observed in medical research. These include: i) Foreign-body giant cells, which are a group of macrophages that form in the presence of large foreign bodies (Ahmadzadeh et al., 2022; Lambert, 1912; Sheikh et al., 2015); ii) Langhans giant cells, which are formed by the fusion of epithelioid cells and contain nuclei arranged in a horseshoe-shaped pattern in the cell periphery (Anderson, 2000; Lay et al., 2007); iii) Touton giant cells, which contain a ring of nuclei surrounding a central homogeneous cytoplasm, while foamy cytoplasm surrounds the nuclei (Dayan et al., 1989); iv) Giant-cell arteritis (Cho et al., 2017); v) Reed-Sternberg cells, which are abnormal lymphocytes found in people with Hodgkin lymphoma (Frierson et al., 1988; Tzankov et al., 2005). **Figure 1** illustrates a schematic representation of the transformation of a macrophage into a multinucleated giant cell (MGC).

It is evident that multinucleation in macrophages occur *in vivo* under chronic inflammatory conditions (Helming and Gordon, 2009; Milde et al., 2015). The fusion of mononuclear phagocytes occurs spontaneously *in vivo* and leads to the differentiation of either multinucleated giant cells or osteoclasts in chronic inflammatory sites or in bone, respectively (Vignery, 2004). Another report showed that macrophage fusion leading to foreign body giant cell formation persists under phagocytic stimulation by microspheres *in vitro* and *in vivo* in mouse models (Jay et al., 2009).

The formation of multinucleated giant cells (MGC) in protozoan parasite *Entamoeba* is mainly observed under conditions of starvation, osmotic stress, or heat stress. However, there have been no reports of MGC formation in other protozoa to date. It is worth noting that other protozoan parasites are capable of inducing MGC formation in human cells. For example, *Leishmania* parasites induce multinucleation of bone marrow-derived macrophages (Hong et al., 2022; Pereira-Neves and Benchimol, 2009).

**Figure 2:**
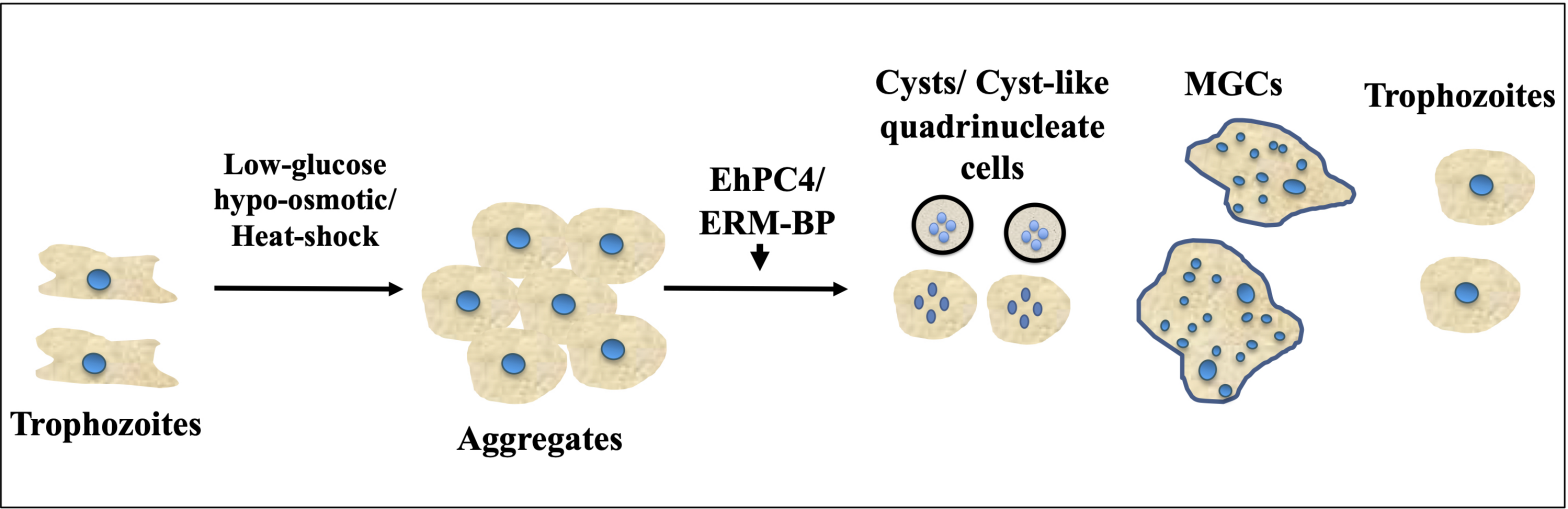
Encystation and heat-shock response triggers formation of MGCs (Multinucleated Giant Cells). Schematic showing different morphological changes during encystation and heat-shock response in *Entamoeba.* Cells form aggregates due to glucose depletion, hypo-osmotic stress or heat-stress. Transcription factor ERM-BP works downstream of aggregate formations and induce MGC formation whereas overexpression of EhPC4 has been shown to increase cell size and lead to the accumulation of multinucleated cells. With the progression of encystation nuclear division takes place to make quadrinucleated cyst or cyst like structure. Cell fusion also involves in low frequency that transform trophozoites into MGC which are highly motile under confinement observed in *Entamoeba*.

In *E. histolytica*, the absence of cell cycle checkpoints results in the separation of nuclear division and cytokinesis, which leads to multinucleation events associated with cytokinesis failure and the termination of ongoing cell division (Das and Lohia, 2002). As a result, *E. histolytica* cells frequently accumulate polyploid cells with a single nucleus as well as multiple nuclei in a single cell, with genome contents ranging from 1X to 10X or more. Furthermore, it has been observed that *Entamoeba* cells can transform into a gigantic cell during the encystation process, which may contain over 200 nuclei. This occurs when motile trophozoites transform into dormant cysts. The formation of multinucleated giant cells (MGC) in *Entamoeba* mainly occurs due to multiple cell fusions triggered by calorie restriction and osmotic stress in the encystation culture media. The cells first form aggregates due to osmotic stress or heat stress and then undergo cell fusion to generate MGC along with quadrinucleated cyst or cyst-like structures, while a few cells remain as single trophozoites (**Figure 2**). The MGCs are highly motile and can continuously undergo cytofission to generate multiple daughter cells when transferred to a new medium or confined. The prevalence of MGCs in encysting cultures is low (1 in 10^4^ cells), and the size of these MGCs varies significantly (Krishnan and Ghosh, 2018; Manna et al., 2020b). At the early stages of encystation, MGCs have been observed to contain nuclei of various sizes, with larger nuclei than trophozoites, as well as collections of tiny nuclei smaller than trophozoite nuclei. As encystation progresses, the number of nuclei in MGCs reduces by half, suggesting that the nuclei undergo haploidization (Krishnan and Ghosh, 2018). Recent studies have also shown that heat stress can lead to the formation of MGCs in *Entamoeba* cells (Manna et al., 2020b). Very little is currently known about the regulators that control the formation of multinucleated cells in *Entamoeba*. However, two transcription factors, EhPC4 and ERM-BP, have been reported to play significant roles in multinucleation and polykaryon formation in *Entamoeba* (Cruz et al., 2016; Manna et al., 2020a). Overexpression of EhPC4 has been observed to significantly increase cell proliferation, DNA replication, and DNA content in trophozoites. Furthermore, overexpression of EhPC4 has been shown to increase cell size and lead to the accumulation of multinucleated cells. This multinucleation phenotype may be due to cytokinesis failure or syncytium formation by cell-cell fusion (de la Cruz et al., 2014). The second factor, ERM-BP, has been reported as a developmentally regulated transcription factor that acts downstream of cellular aggregation and is also an early regulator of development as well as the heat shock response in *Entamoeba* (**Figure 2**). It has also been shown that silencing ERM-BP leads to a significant reduction in MGC formation and the few MGC that do appear are smaller in size (Manna et al., 2020a; Hazra and Manna, 2023).

## Role of multinucleated giant cells (MGCs) in higher eukaryotes

### The occurrence and underlying factors of MGC formation in humans

The formation of MGCs serves different purposes in various biological systems. For example, in dermatology, giant cells are considered to be a significant pathological factor with diagnostic value, although their specific functions remain unclear (Gupta et al., 2014). In terms of pathological conditions, macrophage fusion leads to the formation of multinucleated giant cells along with the foreign body response (FBR) as illustrated in **Figure 1**. Elevated levels of MMP-9 (Matrix Metallo-Proteinase-9) expression have been observed during macrophage fusion, as reported by Sheikh et al. (2015). These giant cells are believed to aid in the defense mechanism of macrophages. To date, only a few surface receptors have been found to be involved in macrophage fusion, such as signal regulatory protein 1-a, IL-4R, E-cadherin, CD44, CD47, CD200, and mannose receptor (MacLauchlan et al., 2009).

Various conditions have been identified to regulate the formation of MGC in culture, including the use of conditioned media or the addition of cytokines, lectins, PMA (phorbol myristate acetate), either alone or in combination with interferon (IFN-γ). Additionally, the formation of MGC can also be facilitated by long-term culture of monocytes *in vitro* (Möst et al., 1997).

The response of macrophage to biomaterials can be schematically depicted based on the size of implanted materials. Macrophages internalize small fragments and particles (<10 μm in diameter) via phagocytosis and intracellular digestion. For particles larger than 10 μm and smaller than 100 μm, macrophages fuse together to form giant cells that engulf and digest the particles (**Figure 1**). For larger particles, macrophages and macrophage-fused giant cells carry out bulk digestion via extracellular degradation by releasing enzymes and/or lowering pH. The formation of multinucleated giant cells involves more than two protoplasts and prevents mitosis and subsequent development (Adapted from Miller et al., 1971 and Sheikh et al., 2015).

### Giant cells in human and their functions

It has been postulated that multinucleated giant cells (MGCs) are discovered first by Paul Langerhans and are formed from the fusion of monocyte-derived macrophages during various inflammatory states in different tissues. MGCs have been found to participate in both pathological and physiological processes (Chambers, 1978; de Jong and Geijtenbeek, 2010).

Recent studies have shed light on the mechanism behind the formation of these giant cells, although it is still not fully understood. It has been proposed that T-helper 2 cytokines, including interleukin 4 (IL-4) and interleukin 13 (IL-13), as well as macrophage fusion receptor, signal-regulatory protein-a (SIRP-a), macrophage colony stimulating factor (M-CSF), granulocyte-macrophage colony-stimulating factor (GM-CSF), IL-17A, interferon-g (IFN-g), receptor activator for nuclear factor-kB ligand (RANKL), cell-specific membrane proteins such as Dendritic cell-specific transmembrane protein (DCSTAMP), transmembrane adhesion receptor cadherin, mannose receptor (such as CD206, which detects microorganisms that express mannose as an oligosaccharide), monocyte chemoattractant protein (MCP1), and integrins (beta 1 and beta 2) all play crucial roles in the fusion process (Brodbeck and Anderson, 2009; Kondo et al., 2009).

Various types of giant cells have been observed in human cells, including Langhans giant cells and Touton giant cells, which are produced by the fusion of macrophage-derived foam cells (Wang and Smart, 2014). MGCs are also found in *Mycobacterium*-induced granulomas, foreign body giant cells (FBGC), osteoclast-like cells, and tumor cells of bone, as well as aneurysmal bone cysts (Gupta et al., 2014; Gupta, 2016; Park et al., 2016). These MGCs exhibit characteristics similar to granulomas and are capable of encountering extracellular materials, such as pathogens and foreign substances.

In humans, the formation of MGCs is associated with various infectious diseases, including tuberculosis, brucellosis, leprosy, and aspergillosis. In an *in vitro* model of human tuberculous granulomas, high-virulence *Mycobacterium* (*Mycobacterium tuberculosis*) led to the formation of large multinucleated giant cells with more than 15 nuclei, while non-pathogenic species of *Mycobacterium* such as *M. avium* and *M. smegmatis* formed smaller cells with fewer nuclei that are not considered MGCs (Kondo et al., 2009). Thus, MGCs play a crucial role in eliminating foreign substances, damaged tissue, and pathogens, which supports host survival.

The osteoclast-like giant cells, also known as osteoclasts, are predominantly bone-resorbing MGCs that play a crucial role in bone remodeling and homeostasis. These cells are derived from bone marrow precursors that originate as early mononuclear macrophages and then circulate in the bloodstream and attach to the bone marrow surface. The process of MGC formation is initiated when the parent Osteoclast cells interact with the osteoclast differentiation factor called Receptor Activator of Nuclear Factor k-B Ligand (RANKL), which in turn promotes the expression of Dendritic cell-specific transmembrane protein and leads to MGC formation. Calcitonin receptor (CTR) and Multinucleation tartrate-resistant acid phosphatase (TRAP) are also available markers used in detecting these osteoclasts. TRAP has a core iron center and is capable of generating ROS (reactive oxygen species), which are involved in bone matrix degradation during antigen presentation and bacterial killing (Halleen et al., 2003).Solberg et al. (2015) have also reported that ROS generated by TRAP plays an essential role in this process.

In the context of bone tissue, giant cell tumors, also known as giant cell myeloma, are benign neoplasms that arise sporadically in long bones. These tumors display phenotypic features similar to osteoclasts. Another type of giant cell, Foreign Body Giant Cells (FBGCs), typically develop as a result of an immunological reaction in patients who use foreign bodies such as catheters. FBGCs contain numerous nuclei, sometimes up to 100, and work to eliminate foreign substances from the host by sequestering the foreign material along with inorganic substances to form an aggregate of endogenous substances. Studies suggest that the formation of FBGCs requires external stimuli and a material surface with appropriate adherent proteins. For example, vitronectin and E-cadherin have been identified as important adhesion proteins during IL-4-induced FBGC formation. *In vitro* models of MGCs derived from monocytes have shown elevated phagocytosis that involves components of endoplasmic reticulum proteins such as calnexin and calregulin, which localize at fusion interfaces with actin and are involved in cell-cell interactions. FBGCs also actively participate in the inflammatory response by producing various cytokines.

There is a correlation between rheumatoid diseases and MGCs, such as rheumatoid arthritis (RA) and rheumatoid heart disease (Kohoutek, 1976). During inflammation in RA patients, the synovial tissue contains an elevated number of macrophages, and the number of MGCs in this tissue is substantially increased. These MGCs have the ability to express tumor necrosis factor (TNF) and interleukin-1 (IL1) (Weinberg et al., 1993). In giant cell arteritis (GCA), a systemic inflammatory innate immune disease, ROS production by multinucleated giant cells and macrophages play an important role in its pathogenesis (Wang et al., 2020).

Sarcoidosis is an autoimmune granulomatous disease that affects the lymphatic systems, as well as the pulmonary and cutaneous systems. It is characterized by the presence of epithelioid cells, macrophages, and multinucleated giant cells. Lymphocytes and fibroblasts may also be present in sarcoidosis. The pathogenesis of this disease is associated with inflammatory cytokines, such as IL-6 and TNF-a (Starshinova et al., 2020).

### Unleashing the impact of MGC on cancer

**Figure 3:**
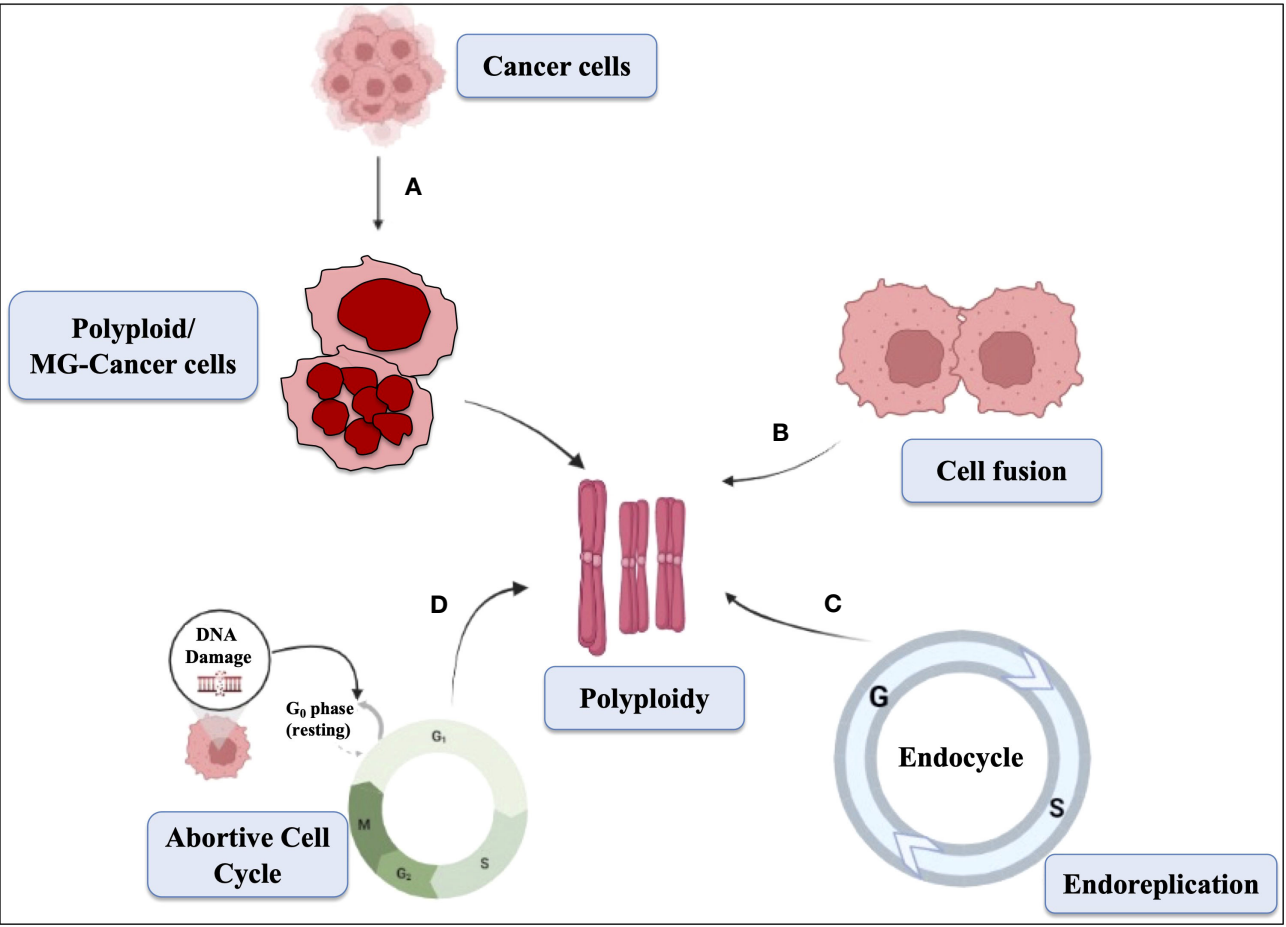
Schematic representation showing the mechanism of polyploid cells. The mechanisms of multinucleation, cell fusion, abortive cell cycle, and endoreplication contribute to the formation of polyploid cells. **(A)** Multinucleation occurs when cells undergo abnormal mitosis, resulting in multiple nuclei within a single cell. **(B)** Cell fusion combines genetic material from different cells, leading to increased chromosome sets. **(C)** Endoreplication involves repeated DNA replication without division. **(D)** Abortive cell cycle disrupts normal progression, causing cells to exit prematurely and generate polyploid cells. These mechanisms collectively drive the development of polyploidy in cells, which can promote genomic instability, tumor progression, and resistance to treatment in cancer cells.

Polyploid giant cells, which often have multiple nuclei, have been observed in tumors and cell lines derived from tumors. Polyploidy is not only associated with cancer, but is also seen in aging and diabetes. In diploid organisms, polyploid cells can form via three general mechanisms, including cell fusion, endoreplication, and a variety of defects that result in an abortive cell cycle (as shown in **Figure 3**). It has been speculated that polyploid giant tumor cells may facilitate rapid tumor evolution and the acquisition of therapy resistance in multiple incurable cancers (Coward and Harding, 2014). Polyploidy seems to provide cancer cells with adaptability in harsh conditions such as low serum, hypoxia, and exposure to drugs. There is a strong correlation between the generation of polyploid cells and various cellular stressors, and catastrophic DNA replication has been observed in unscheduled polyploid cells (Manic et al., 2022). The formation of polyploid giant cancer cells (PGCCs), which are responsible for genomic instability and chemotherapy resistance, occurs under the pressure of chemotherapy and results in cells with more chromosomes than diploid cells (Zhou et al., 2022).

The generation and maintenance of polyploid cells have been the subject of limited research, and the exact mechanisms involved remain unclear. In ovarian cancer cell lines, it was observed that Autophagy inhibitors did not prevent the formation of polyploid giant cancer cells (PGCCs) (Bowers et al., 2022). Similarly, Rapamycin did not induce PGCC formation on its own, nor did it exacerbate PGCC formation brought on by chemotherapy. A recent study established a link between latent PGCCs and the clinical risk of nasopharyngeal carcinoma (NPC) recurrence (Chen et al., 2022). The study demonstrated that mitochondrial changes and chemotherapeutic medications that cause partial mitochondrial damage led to low ATP levels and autophagy activation through the AMPK-mTOR pathway, which in turn helps generate PGCC. **Figure 3** shows several mechanisms responsible for polyploidy generation. The reversibility of proliferative arrest, which is often associated with the combination of cell senescence with polyploidization/depolyploidization, appears to be crucial to this process (Saleh et al., 2022). The study suggests that progeny resulting from polyploid cells reverting back to the euploid state through a reversal process could be highly aggressive and lead to the generation of resistant tumors. However, the role of polyploidy in senescence and how polyploid cells that exhibit senescence revert back to normal remain unknown.

### Role of MGC in plants

Plants, like animals, also have the ability to generate MGCs. Root Knot Nematodes (RKN) of the *Meloidoyne spp* obligate parasite have been reported to infect plants by inducing the redifferentiation of root cells into multinucleated and hypertrophied feeding cells, which are known as giant cells. These giant cells arise due to repeated rounds of karyokinesis without cell division (Antonino de Souza Junior et al., 2017; Meidani et al., 2019). Studies have shown that Microtubule-Associated Protein-65-3 (MAP65-3) in *Arabidopsis thaliana* is crucial for the ontogenesis of giant cells in plant-nematode interactions. In the absence of functional MAP65-3, cytokinesis was initiated but not completed in these giant cells, indicating that MAP65-3 plays a major role in giant cell development (Caillaud et al., 2008).

The initiation of giant cell formation during sepal development in *Arabidopsis* is regulated by the fluctuation of the transcription factor ATML1, a homeobox gene of *Arabidopsis*. Studies have revealed that the suppression of ATML1 up to a threshold level during G2 phases of the cell cycle is highly likely to lead to giant cell establishment and entry into endoreduplication (Meyer et al., 2017). Another transcription factor of *Arabidopsis thaliana*, PUCHI, has also been found to regulate giant cell morphology during gall development (Suzuki et al., 2021).

## Role of MGC in parasites

### A fascinating look into giant protozoa

Recent research on the composition of zooplankton has shed new light on the importance of giant protozoa in ocean ecosystems. Among the various types of protozoa, only a limited number exhibit a multinuclear structure. *Pelomyxa*, for instance, belongs to a genus of flagellar amoebae that are characterized by their large size and possession of multiple nuclei. Another genus in the Amoebidae family, *Chaos*, is also known for its members’ enormous size, with *Chaos carolinensis* being recognized as a giant amoeba.

Protozoan parasites have been extensively studied with respect to their giant cells during the formation of cysts in *Entamoeba* species. This occurs due to continuous cell fusion and division, leading to the aggregation of haploid nuclei and the formation of a polyploid nucleus. Moreover, polyploidy can occur without nuclear division, resulting in the accumulation of several genome contents in each nucleus of a single cell, which indicates that DNA reduplication occurs multiple times before cell division and contributes to the formation of giant cells (Das and Lohia, 2002).

*E. invadens*, a pathogen found in reptiles, has been extensively utilized as a model organism to investigate encystation events. However, recent studies have revealed that *E. histolytica*, causing amoebiasis to human, is also capable of forming cysts under *in vitro* conditions (Wesel et al., 2021). This newfound ability adds a new dimension to our understanding of *E. histolytica’s* lifecycle and opens avenues for further research in this area. It has been found that multinucleated giant cells (MGC) aggregate in encystation culture when trophozoite cells are exposed to any stressful condition (Krishnan and Ghosh, 2018).

### Formation of giant cell occurs naturally

The presence of multinucleation in *E. histolytica* has been demonstrated through *in vivo* observations (Mukherjee et al., 2008). Notably, staining of human intestinal tissue sections from cases of amoebic colitis has provided compelling evidence of this phenomenon. In these stained tissue sections, the characteristic multinucleated cells of *E. histolytica* are readily identifiable (Mukherjee et al., 2008). No only *Entamoeba*, a new type of amoeba called *Xenophyophore* has recently been discovered, which is the largest known multinucleated unicellular organism to date (Gooday et al., 2020). The average size of these *Xenophyophore* is approximately 10 cm, but some may grow up to 20 cm. In 2011, a team of marine biologists from the Scripps Institute of Oceanology conducted a search in the dark depths of the Mariana Trench, and to their surprise, they discovered this unicellular giant organism at a depth below 10,500 meters below sea level (Gooday et al., 2020).

It is debatable whether these *Xenophyophore* can be classified as amoeba or not. Initially, when they were discovered, scientists believed they were a variety of sponges. Later, it was determined that they were a type of gigantic amoeba, as they move with the help of pseudopods. However, some researchers now classify them as part of the foraminifera group, as these giant cells have a shell-like structure around them. Interestingly, the foraminifera group of organisms are amoeba-like protists that are unicellular in nature, but are also known as “armoured amoebae” due to their shells. These cells acquire minerals from sea sediment, which makes them resistant to the toxic effects of many heavy metals (Koonce et al., 1986; Rieu et al., 2015).

### Fate of giant *Entamoeba* cells

Encystation is a primitive survival process used by all Amoebozoa (Schaap and Schilde, 2018; Yoshida, 1918). Cysts can be asexual or sexual, with zygotes formed through cell fusion eventually undergoing encystation (Brunet and King, 2017). In a similar manner, MGCs are formed inside cell aggregates, with initial cell signaling cooperating with encystation. The expression of meiotic genes may lead to some cells gaining fusion competency, facilitating the MGC pathway.

The formation of multinucleated giant cells (MGC) is a survival mechanism employed by *Entamoeba* to cope with unfavorable environmental conditions like starvation, osmotic stress, and heat stress. The fusion of multiple cells to form MGC enables them to withstand and overcome these stressors. Additionally, when these giant cells were transferred to a nutrient-rich medium under confinement, meiosis occurred, and they were able to divide and transform into smaller trophozoites. This indicates that MGC formation is an intermediate stage for the survival of *Entamoeba* in adverse conditions.

Cell fusion and meiosis, which allow genetic recombination during encystation, are believed to act as survival mechanisms allowing cells to remain dormant as cysts or as polyploid MGCs (Cavalier-Smith, 2010; Tekle et al., 2017). Although MGC cell fusion occurs asexually in *Entamoeba*, the study of its cellular activities could be a useful parameter for understanding the origin and evolution of sexual reproduction, as this organism is considered a primitive eukaryote (Bakker-Grunwald and Wöstmann, 1993).

**Figure 4:**
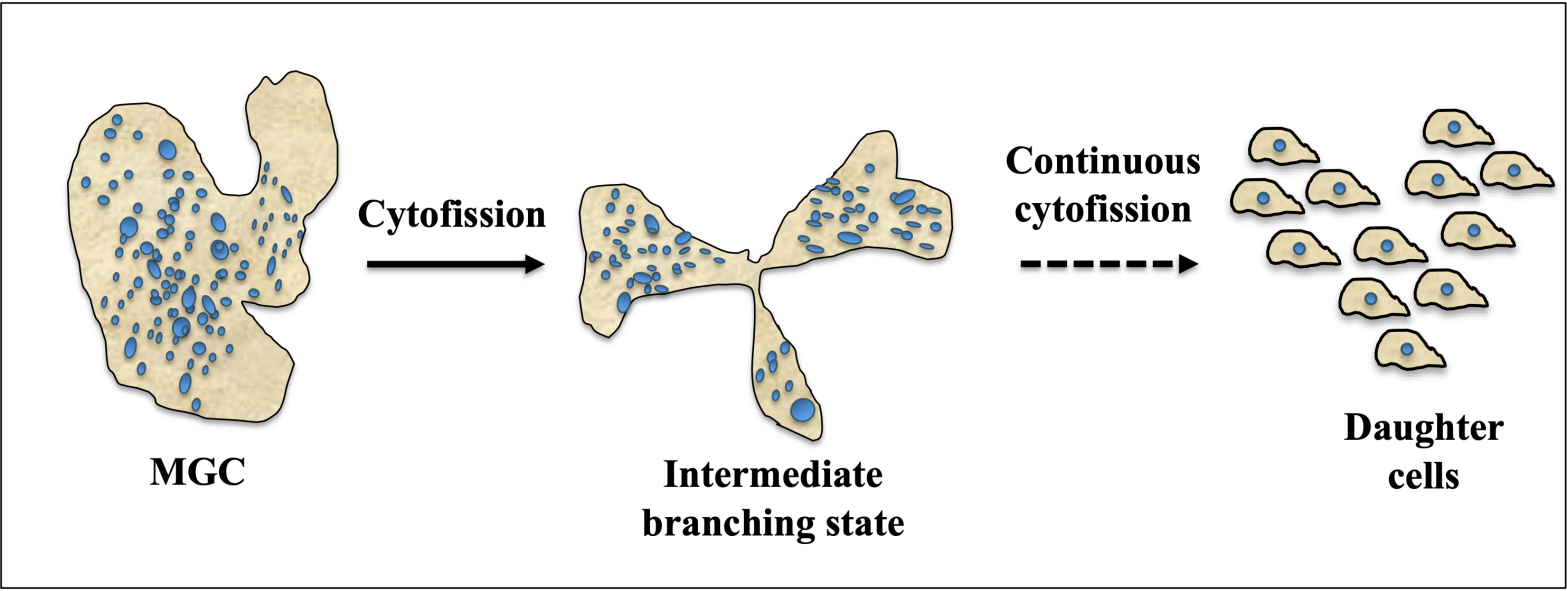
Cytofission in MGC (Multinucleated Giant Cell). Under favorable conditions, MGCs exhibit a unique behavior where they move in multiple directions and undergo a process of branching. This intermediate branching state allows the MGCs to divide and separate into smaller cells. The intermediate cells resulting from this branching are capable of continuous cytofission, a process involving the division of the cytoplasm, until they reach the size of trophozoite daughter cells. This continuous division and separation into smaller cells enable the MGCs to proliferate and generate a population of trophozoite-sized daughter cells, potentially contributing to the expansion and dissemination of the MGC population.

Cytofission in *E. invadens* starts randomly and daughter cells continue to divide until they reach trophozoite size, as shown in **Figure 4**. The distribution of nuclei during cytofission is uneven, and the arrangement of nuclei into daughter cells is detected with the help of the cell-permeable nuclear stain Hoechst33342. However, no correlation was found between the number of nuclei per cell and daughter cell size (Krishnan and Ghosh, 2018). Cytoplasmic bridges connecting daughter cells were observed in the area where nearby trophozoites converge, suggesting their occurrence during *E. invadens* cell division (Biron et al., 2001).

Although there are limited reports on whether amoebic giant cells are more phagocytic than trophozoites, studies have shown that the phagocytic property of giant macrophages increases. Specifically, reports have indicated increased phagocytosis of macrophages towards specific cells or molecules that are opsonized by the complement system (Sheikh et al., 2015). It has been reported that macrophage MGCs can effectively engulf larger particles that single macrophages cannot (Milde et al., 2015). Additionally, these cells have been observed to consume cellular debris and waste from injured tissues and healing sites. These multinucleated cells are also known to be involved in complement-mediated phagocytosis and the destruction of large targets (Jay et al., 2009).

### Initial giant cells are more competent for fusion

The motile amoeba trophozoites exhibit strong adhesion to surfaces and move actively via their pseudopods. In contrast, giant cells do not adhere and instead float randomly in encystation media. When two of these non-adherent cells come into contact, they fuse and give rise to a larger giant cell. According to the collected data so far, the initial giant cells appear to be more competent and prone to fusion with other cells, resulting in the formation of even larger cells (Manna et al., 2020b). However, after 72 h of encystation giant cells in the media are less engaged in such fusions and tend to move or float randomly (Krishnan and Ghosh, 2018; Manna et al., 2020b). Compared to trophozoites, giant cells exhibit slow movement, likely due to their larger size and diameter. However, when confined or subjected to external pressure or mechanical stress, these giant cells demonstrate increased motility. It appears that these giant cells attempt to escape the situation by any means possible, resulting in the fragmentation of the cells into smaller pieces (Krishnan and Ghosh, 2018) To be more specific, under confinement, multinucleated giant amoeba cells undergo binary fission. Additionally, the giant cells do not appear to fuse with normal trophozoites, suggesting the possibility of the presence of a specific surface protein unique to these competent cells. But the genome of *Entamoeba* does not contain any sequence for any known protein responsible for fusion. So exactly what factors are aiding in the fusion competency is still unknown (Krishnan and Ghosh, 2018).

## Concluding and future perspective

Every organism possesses unique strategies to adapt to environmental challenges, and the protozoan parasite *Entamoeba* exemplifies this adaptability. In the case of the protozoan parasite *Entamoeba*, it has a stage known as the ‘trophozoite’ that thrives and reproduces in favorable growth conditions. However, in adverse conditions, it transforms into a ‘cyst’ stage through a process called encystation. This cyst is shielded by a thick chitin wall and can endure extreme environmental conditions like high temperature, pressure, and lack of food. *Entamoeba* also utilizes an alternative survival pathway by forming multinucleated giant cells (MGCs) through cell fusion (Bracha and Mirelman, 1984; Mukherjee et al., 2008). Research has shown that during encystation, cells fuse and give rise to MGCs (Bos, 1975; Krishnan and Ghosh, 2018). Signaling within cell aggregates plays a critical role in encystation and MGC formation, however, the precise mechanistic pathways of MGC formation are unclear (Krishnan and Ghosh, 2018; Manna et al., 2020b). It is possible that individual cells unite to survive and acquire advantageous traits from multinucleated cells. In cancer cells, cell fusion leads to genome reorganization and metastasis, and a similar phenomenon may induce phenotypic variations in *Entamoeba*. Encystation ensures survival in adverse conditions by forming resilient cysts, while the MGC pathway may introduce beneficial changes for survival. MGC formation alters cellular machinery and ploidy, potentially impacting *Entamoeba’s* ability to survive and causing inflammatory reactions in the human body. The conjecture that polyploid or MGC *Entamoeba* may exhibit heightened pathogenicity or infectivity requires further investigation. The future perspectives would be- investigating the mechanistic pathways of MGC formation, exploring the role of MGCs in phenotypic variations, unraveling the impact of MGCs on pathogenicity and infectivity, unraveling the impact of MGCs on pathogenicity and infectivity and characterizing the genetic and genomic changes associated with MGC formation.

## Author contributions

SH, SD, NBK, SRH, PD, AM, PM, and DM wrote the main manuscript. SH, SD, PM, and DM prepared the figures. All authors contributed to the article and approved the submitted version.
